# Associations between GoSmart Channel, health literacy and health behaviours in adolescents: A population‐based study

**DOI:** 10.1111/hex.13894

**Published:** 2023-10-26

**Authors:** Junjie Huang, Sze Chai Chan, Vera M. W. Keung, Calvin K. M. Cheung, Amelia S. C. Lo, Vincent T. C. Lau, Lancelot W. H. Mui, Albert Lee, Martin C. S. Wong

**Affiliations:** ^1^ Centre for Health Education and Health Promotion, Faculty of Medicine The Chinese University of Hong Kong Hong Kong Hong Kong SAR; ^2^ Jockey Club School of Public Health and Primary Care The Chinese University of Hong Kong Hong Kong Hong Kong SAR; ^3^ The School of Public Health Peking University Beijing China; ^4^ The School of Public Health The Chinese Academy of Medical Sciences and The Peking Union Medical Colleges Beijing China; ^5^ The School of Public Health Fudan University Shanghai China

**Keywords:** adolescents, health behaviour, healthy literacy

## Abstract

**Background:**

Health literacy is essential in the promotion of healthy lifestyle habits and chronic disease prevention.

**Objective:**

To assess the health literacy level among Hong Kong adolescents; to evaluate the association between access of an online health information platform (GoSmart Channel) and health literacy level; and to examine the association between health literacy level and various unhealthy behaviours.

**Design:**

This study recruited students from 10 local secondary schools in Hong Kong to assess the health literacy level among adolescents.

**Settings and Participants:**

Participants were required to complete a self‐administered questionnaire on health behaviours and health literacy using the Health Literacy Measure for Adolescents (HELMA).

**Main Outcome Measure:**

Data were analysed using descriptive statistics and multivariate regression modelling.

**Results:**

A total of 777 responses were collected. Overall, most (74.4%) of the adolescents in Hong Kong have limited health literacy (HELMA score <66). The majority (63.7%) of adolescents relied on their parents for health information, while 11.4% of the respondents sought information from the GoSmart Channel. The intervention of GoSmart Channel was significantly associated with better health literacy in almost all aspects among adolescents. Desired levels of health literacy were significantly associated with better perceived health (adjusted odds ratio: 2.04, *p* = .001) and negatively associated with a range of unhealthy and risky behaviours including unhealthy dietary habits, poor hygienic measures and physical inactivity.

**Discussion and Conclusion:**

This study highlights the importance of improving health literacy among Hong Kong adolescents and the potential of technology‐based interventions. The findings suggest the need for continued efforts to promote health literacy and healthy behaviours among adolescents, especially given the limited health literacy levels observed in the study.

**Patient or Public Contribution:**

Members of the GoSmart.Net Built‐on Project patient and public involvement and engagement group advised about survey development.

## 1 INTRODUCTION

Adolescent health literacy is essential in the promotion of healthy lifestyle habits and chronic disease prevention. Health literacy refers to the personal knowledge and competencies accumulated through various means to access, understand, appraise and use information and services in ways that promote and maintain good health and wellbeing.^1^ Low health literacy has been associated with a range of unhealthy behaviours, including poor dietary habits, physical inactivity, risky sexual behaviours and substance abuse.^2^, ^3^ Studies have shown that most of these behaviours are developed during adolescence. For instance, adolescents engaged in obesogenic behaviours had a greater risk for obesity and obesity‐related health complications in adulthood.^4^, ^5^ Despite efforts to improve adolescent health literacy,^6^ studies have shown that nearly half of the adolescents in Taiwan and some European countries have insufficient or problematic health literacy skills, especially in the domain of health information appraisal.^2^, ^7^, ^8^

With the aim to enhance adolescents' knowledge of health‐related topics, improve their ability to access and understand health information and enhance their decision‐making skills related to health behaviours, ‘GoSmart Channel’, a health promotion initiative, was implemented by our research team.^9^ Through collaborative efforts with multiple local primary and secondary schools, the online platform aims to produce high‐quality health education videos covering a wide spectrum of health and wellbeing and optimize teaching resources in diverse formats.

Although adolescent health literacy is a crucial part of health promotion, it received far less attention than adults' health literacy.^6^, ^10^ To our knowledge, there has been little research on this topic in a secondary school setting in Hong Kong, while existing literature focused on oral^11^ and mental health literacy. This study aims: (1) to assess the health literacy level among Hong Kong adolescents; (2) to evaluate the association between access of GoSmart Channel and health literacy level; (3) and to evaluate the association between health literacy level and different unhealthy behaviours.

## 2 METHODOLOGY

### 2.1 Description on the project

Our research team has collaborated with teacher coordinators from 18 local secondary schools in various districts of Hong Kong. We have received the school's endorsement to participate in the GoSmart.Net Built‐on Project. After the recruitment of participating secondary schools, teachers were contacted for needs assessment and a working group of teachers was formed to support project implementation. Thirty videos were uploaded to GoSmart Channel at the beginning of the project, while 20 school‐based videos were made and uploaded with support from the research group. Meanwhile, student training seminars on health promotion were conducted.

### 2.2 Sampling method

Since there was no previous study done in Hong Kong, a confidence level of 99% and a margin of error of approximately 5% were assumed. The formula $n=\frac{Z^{2}\times p\times (1-p)}{E^{2}}$ was used. Assuming a confidence level of 99% (*Z* = 2.58), a probability of positive outcomes of 50% (*p* = .5) and a nonresponse rate of 10%, the required sample size would be approximately 732 to achieve a margin of error of approximately 5% with a confidence level of 99%. Eligible participants were students from the secondary school participating in the GoSmart.Net Built‐on Project. There were no exclusion criteria and age limit for the study, and all eligible participants were included in the analysis.

### 2.3 Ethics

A letter of invitation was sent to the school principal of each participating secondary school to explain the purpose of a survey aimed at evaluating the effectiveness of a project. Upon obtaining the school's consent, hard copies of questionnaires were distributed to each participating secondary school. In accordance with ethical guidelines, school consent is sufficient for the invitation of secondary school students to participate in an anonymous general questionnaire. However, the team strongly recommends obtaining parental consent as an additional measure. To ensure the voluntary nature of participation, the research team has advised school teachers to emphasize this aspect when inviting students to complete the survey. Students were also clearly informed that they were allowed to leave some questions blank if they were not willing to answer them.

### 2.4 Survey instrument

Participants were asked to complete a self‐administered questionnaire on health behaviours and health literacy. The questionnaire consists of four main parts with a total of 57 questions, including: demographic information (sex, age and socioeconomic status [SES]) and health literacy, source of health information and lifestyle habits and health behaviours (unhealthy dietary, screen time, physical activity level, personal hygiene, etc.). The detailed questionnaire can be found in Material S2. The Family Affluence Scale (FAS) was used as a proxy to measure the SES of children and adolescents, as obtaining accurate information regarding parental SES from them is difficult due to the lack of knowledge and unwillingness to report relevant information, with good internal consistency and test–retest reliability.^12^, ^13^ The scale includes questions on ownership of private bedrooms, ownership of cars (households), number of household computers and travelling history in the past 12 months. A composite score was calculated based on the answers of these four items: low FAS (score = 0–3), medium FAS (score = 4, 5) and high FAS (score = 6, 7) indicated low, medium and high affluence, respectively.^13^

The Health Literacy Measure for Adolescents (HELMA) is a tool that is used to measure health literacy in adolescents, with good internal consistency and test–retest reliability.^2^, ^14^ The HELMA questionnaire consists of 44 items that assess various aspects of health literacy, including knowledge, motivation and self‐efficacy related to health behaviours. Responses were scored on a 5‐point Likert scale, with higher scores indicating higher levels of health literacy.^14^ The raw scores were added and linearly transferred to a score from 0 to 100 for the calculation of the scores overall and each subscale. The overall score and scores of each of the subscales were classified into four categories: adequate [0–50], problematic [50–66] (which together defines limited health literacy), sufficient [66–84] and excellent [84–100] (which together defines desired health literacy). The number of items and the corresponding questions for each subscale are listed in Material S1. The questionnaire was translated into Chinese and back‐translated by a native speaker for validation. The questionnaire is also available in English for those students who can only read English.

### 2.5 Statistical analysis

All data were entered and analysed using the IBM Statistical Package for Social Sciences (SPSS) software version 26.0. Descriptive analyses were conducted to summarize participant characteristics and HELMA scores. Mean scores and standard deviations were calculated for each subscale of the HELMA questionnaire. The association between access of GoSmart Channel and desired health literacy and between desired health literacy and healthy lifestyle habits and behaviours were evaluated using multivariate logistic regression modelling. Statistical significance was set at .05.

## 3 RESULTS

### 3.1 Participant characteristics

A total of 777 responses were collected and the participants' mean age was 13.57 years (SD: 1.16) (Table 1). Among them, 48.4% (*n* = 376) were male, and the majority of them (74.9%, *n* = 573) were from families with low SES. In addition, 60.9% (*n* = 471) of the respondents reported that their household did not own a car, while half of them (50.1%, *n* = 387) had their own bedroom. Furthermore, 93.5% (*n* = 722) did not leave Hong Kong for travel in the past 12 months, while 74.4% (*n* = 575) had two or more computers at home.

**Table 1:** Participant characteristics.

	Mean (SD)/*n* (%)
Age (mean, SD)	13.57, 1.16
Sex	
Female	401 (51.6%)
Male	376 (48.4%)
Family Affluence Scale status	
Low	573 (74.9%)
Medium	177 (23.1%)
High	15 (2.0%)
Ownership of cars (household)	
No	471 (60.9%)
1	213 (27.5%)
2 or more	90 (11.6%)
Ownership of private bedroom	
No	386 (49.9%)
Yes	387 (50.1%)
Travelling (abroad) in the past 12 months	
No	722 (93.5%)
Once	29 (3.8%)
Twice or more	21 (2.7%)
Ownership of computers (household)	
No	33 (4.3%)
1	165 (21.3%)
2 or more	575 (74.4%)
Overall health (self‐perceived)	
Poor to average	226 (29.1%)
Good	550 (70.9%)
Poor hygiene practice	
No	505 (68.2%)
Yes	235 (31.8%)
Intake of vegetables	
Insufficient	644 (85.8%)
Sufficient	107 (14.2%)
Intake of fruits	
Insufficient	353 (47.3%)
Sufficient	393 (52.7%)
Skipping breakfast	
No	403 (57.2%)
Yes	302 (42.8%)
Any physical activity per week	
No	150 (20.0%)
Yes	601 (80.0%)
Time spent on TV	
Less than 2 h	251 (33.8%)
2 h or more	492 (66.2%)
Time spent on games	
Less than 2 h	340 (46.1%)
2 h or more	398 (53.9%)
Time spent on social media	
Less than 2 h	419 (56.4%)
2 h or more	324 (43.6%)
Smoking in the past 30 days	
No	715 (96.9%)
Yes	23 (3.1%)
Alcohol drinking in the past 30 days	
No	642 (87.5%)
Yes	92 (12.5%)

The results showed that the majority of the respondents reported good perceived health (70.9%, *n* = 550) and good hygiene practices (68.2%, *n* = 505). On the other hand, 85.8% (*n* = 644) and 52.7% (*n* = 393) reported insufficient intake of vegetables and fruits, respectively. In addition, 42.8% (*n* = 302) reported skipping breakfast at least once a week, and 20.0% (*n* = 150) reported having no physical activity at all. A significant proportion of respondents spent more than 2 h on watching television (66.2%, *n* = 492), playing games (53.9%, *n* = 398) and using social media (43.6%, *n* = 324). Lastly, a small percentage of the respondents reported smoking (3.1%, *n* = 23) and consuming alcohol (12.5%, *n* = 92) in the past 30 days.

### 3.2 Source of health information

Overall, relatives were the most commonly reported source of health information (63.7%, *n* = 482), followed by school programmes on health‐related topics (56.2%, *n* = 426), TV programmes or other videos (55.3%, *n* = 417) and social media platform (48.6%, *n* = 368) (Supporting Information S1: Table 1). Meanwhile, the GoSmart Channel was utilized as a source of health information by 11.4% (*n* = 86) of the respondents.

### 3.3 Level of health literacy among respondents

In general, health literacy was limited among respondents (Supporting Information S1: Table 2a,b). The overall (74.4%; mean: 54.98, SD: 17.39) and the mean scores for all subscales were categorized into ‘problematic’ (45.8%–78.0%; mean: 51.82–60.68). Respondents had the highest scores on the subscales of understanding (proportion of desired level: 41.1%; mean: 60.68, SD: 21.33), numeracy (54.2%; mean: 59.95, SD: 33.98), appraisal (33.7%; mean: 57.59, SD: 21.65); and the lowest scores on communication (22.0%; mean: 51.82, SD: 21.09), self‐efficacy (22.7%; mean: 51.85, SD: 20.42) and use (25.8%; mean: 52.06, SD: 22.64).

Respondents from the GoSmart Channel group had a significantly higher overall health literacy score (61.29 vs. 54.21, *p* < .001; Supporting Information S1: Table 2a). Among all subscales except numeracy, respondents from the GoSmart Channel group had a higher score of about 8%–25% than the control group. The most significant differences were observed in the subscale of communication (62.35 vs. 50.40, *p* < .001) and use (61.41 vs. 50.71, *p* < .001).

**Figure 1:**
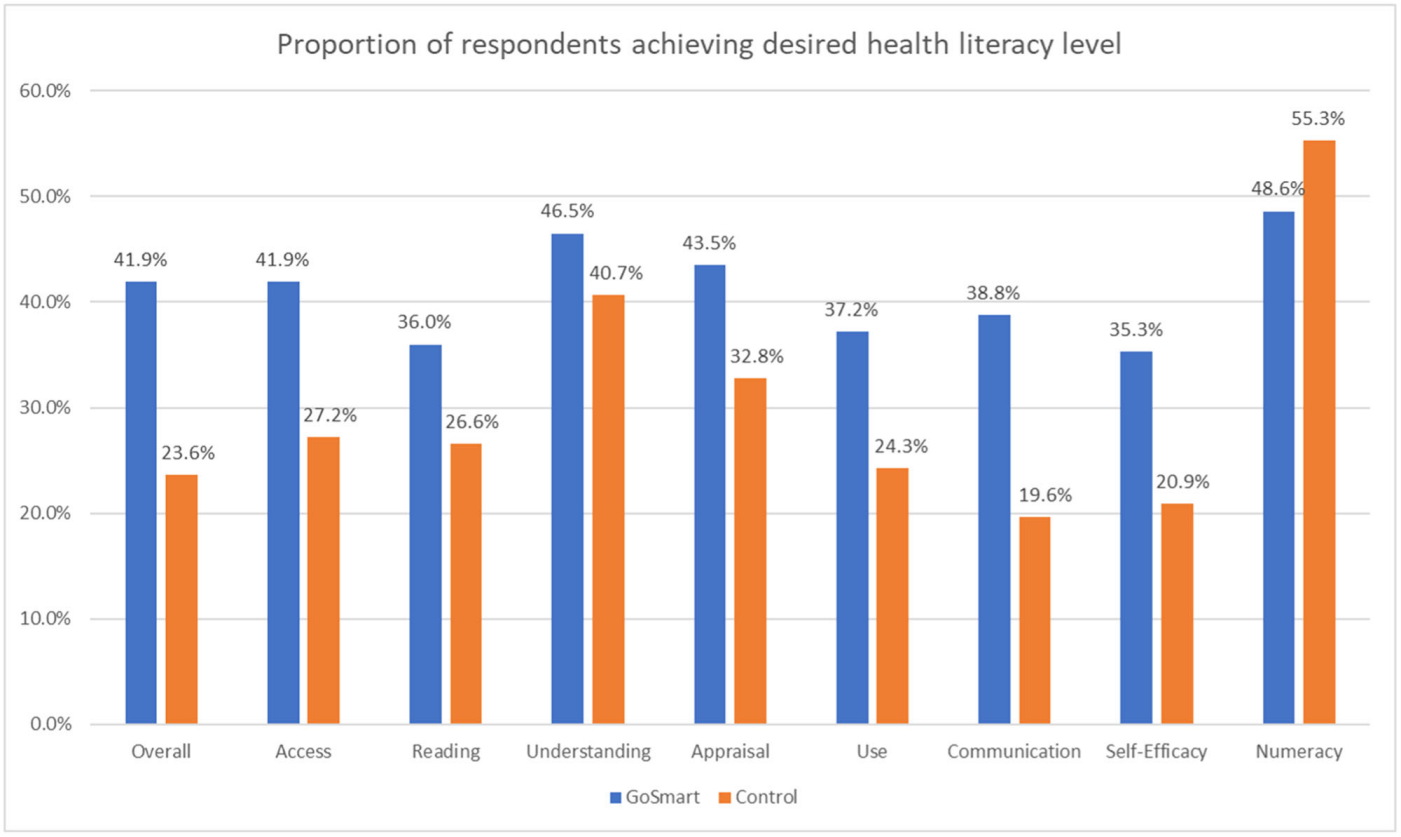
Proportion of respondents achieving desired health literacy level.

Adjusted for sex and SES, respondents from the GoSmart Channel group were significantly more likely to achieve desired health literacy (41.9% vs. 23.6%; adjusted odds ratio [aOR]: 2.45, 95% confidence interval [C]I: 1.53–3.91, *p* < .001) overall than respondents from the control group (Figure 1, Table 2 and Supporting Information S1: Table 2b). Associations were also found between access of GoSmart Channel and desirable level of health literacy in the subscales of access (aOR: 2.03, 95% CI: 1.27–3.24, *p* = .003), reading (aOR: 1.61, 95% CI: 1.001–2.60, *p* = .049), appraisal (aOR: 1.64, 95% CI: 1.03–2.61, *p* = .036), use (aOR: 1.85, 95% CI: 1.15–2.98, *p* = .011), communication (aOR: 2.61, 95% CI: 1.61–4.22, *p* < .001) and self‐efficacy (aOR: 2.12, 95% CI: 1.30–3.46, *p* = .002).

**Table 2:** Factors associated with desired level of health literacy.

	GoSmart Channel (users vs. nonusers [reference group])		Gender (female vs. male [reference group])	
Overall (aOR^a^)	**2.449 (1.533–3.914)**	***p*** < .001*	0.950 (0.679–1.330)	*p* = .767
Access	**2.030 (1.274–3.235)**	***p*** = .003*	1.196 (0.867–1.651)	*p* = .276
Reading	**1.613 (1.001–2.597)**	***p*** = .049*	1.005 (0.726–1.391)	*p* = .978
Understanding	1.310 (0.831–2.066)	*p* = .245	0.924 (0.687–1.244)	*p* = .604
Appraisal	**1.642 (1.034–2.607)**	***p*** = .036*	1.062 (0.781–1.445)	*p* = .701
Use	**1.851 (1.150–2.980)**	***p*** = .011*	0.789 (0.565–1.101)	*p* = .163
Communication	**2.609 (1.611–4.224)**	***p*** < .001*	0.724 (0.507–1.035)	*p* = .077
Self‐efficacy	**2.123 (1.304–3.455)**	***p*** = .002*	0.766 (0.540–1.086)	*p* = .135
Numeracy	0.758 (0.459–1.252)	*p* = .279	1.062 (0.775–1.457)	*p* = .279

	FAS score (medium vs. low [reference group])		FAS score (high vs. low [reference group])	
Overall (aOR^a^)	1.066 (0.719–1.582)	*p* = .750	**4.485 (1.521–13.229)**	***p*** = .007*
Access	0.935 (0.637–1.374)	*p* = .733	2.105 (0.715–6.203)	*p* = .177
Reading	1.066 (0.727–1.564)	*p* = .742	**3.821 (1.298–11.252)**	***p*** = .015*
Understanding	0.919 (0.646–1.307)	*p* = .638	2.548 (0.840–7.735)	*p* = .099
Appraisal	0.941 (0.653–1.357)	*p* = .746	2.074 (0.714–6.028)	*p* = .180
Use	1.149 (0.781–1.692)	*p* = .480	0.831 (0.227–3.038)	*p* = .779
Communication	1.167 (0.773–1.761)	*p* = .463	1.585 (0.484–5.189)	*p* = .446
Self‐efficacy	1.008 (0.668–1.521)	*p* = .970	2.725 (0.921–8.062)	*p* = .070
Numeracy	1.125 (0.771–1.642)	*p* = .542	0.347 (0.089–1.362)	*p* = .129

*Note*: Bold values are statistically significant *p* < 0.05.

Abbreviations: aOR, adjusted odds ratio; FAS, Family Affluence Scale.

^a^ aORs are adjusted for the use of GoSmart Channel, gender and FAS score.

*p < 0.05.

### 3.4 Association between health literacy and health‐related behaviours

A higher proportion of respondents with desirable health literacy reported good health than those with limited health literacy (81.1% vs. 67.6%, *p* < .001) (Supporting Information S1: Table 3). Meanwhile, a smaller proportion of respondents with desirable health literacy reported unhealthy behaviours including poor hygiene (21.2% vs. 35.5%, *p* < .001), insufficient intake of vegetables (77.5% vs. 88.7%, *p* < .001) and fruits (43.1% vs. 56.0%, *p* = .002), skipping breakfast (34.9% vs. 45.6%, *p* = .012), physical inactivity (14.2% vs. 22.2%, *p* = .018) and spending more than 2 h on TV (59.8% vs. 68.6%, *p* = .027), games (43.3% vs. 57.7%, *p* = .001) and social media (36.4% vs. 46.5%, *p* = .016).

Similarly, results from multivariate logistic regression analyses (Table 3) showed that desirable health literacy was positively associated with self‐perceived good health (aOR: 2.04, 95% CI: 1.36–3.04, *p* = .001) and negatively associated with poor hygiene (aOR: 0.49, 95% CI: 0.33–0.72, *p* < .001), insufficient intakes of vegetables (aOR: 0.43, 95% CI: 0.28‐0.67, *p* < .001) and fruits (aOR: 0.58, 95% CI: 0.4–1–0.81, *p* = .002), skipping breakfast (aOR: 0.64, 95% CI: 0.45–0.91, *p* = .012), physical inactivity (aOR: 0.56, 95% CI: 0.35–0.90, *p* = .016) and spending more than 2 h on TV (aOR: 0.67, 95% CI: 0.48–0.95, *p* = .026), games (aOR: 0.54, 95% CI: 0.39–0.76, *p* < .001) and social media (aOR: 0.66, 95% CI: 0.46–0.93, *p* = .019), after adjusting for sex and SES. As far as substance abuse is concerned, respondents with desired health literacy were marginally significantly less likely to have smoked (aOR: 0.13, 95% CI: 0.02–1.01, *p* = .051) in the past 30 days. Meanwhile, respondents with desired health literacy were associated with a lower prevalence of alcohol consumption in the past 30 days (aOR: 0.64, 95% CI: 0.37–1.12, *p* = .120). However, the association was not statistically significant.

**Table 3:** Associations between health literacy level and health behaviours.

	Health literacy^a^ (desirable vs. limited [reference group])		Gender^a^ (female vs. male [reference group])	
Overall good health (self‐perceived)	**2.036 (1.363–3.041)**	***p*** = .001*	1.056 (0.768–1.452)	*p* = .736
Poor hygiene	**0.485 (0.327–0.719)**	***p*** < .001*	0.790 (0.575–1.085)	*p* = .145
Insufficient intake of vegetables	**0.433 (0.279–0.671)**	***p*** < .001*	0.752 (0.490–1.154)	*p* = .192
Insufficient intake of fruits	**0.580 (0.413–0.812)**	***p*** = .002*	0.891 (0.664–1.195)	*p* = .441
Skipping breakfast	**0.637 (0.448–0.906)**	***p*** = .012*	1.331 (0.981–1.806)	*p* = .066
Physical inactivity	**0.563 (0.353–0.899)**	***p*** = .016*	**2.018 (1.380–2.950)**	***p*** < .001*
Spending >2 h on TV	**0.674 (0.476–0.954)**	***p*** = .026*	1.109 (0.812–1.515)	*p* = .514
Spending >2 h on games	**0.544 (0.387–0.764)**	***p*** < .001*	0.765 (0.568–1.031)	*p* = .078
Spending >2 h on social media	**0.656 (0.462–0.933)**	***p*** = .019*	**2.145 (1.585–2.901)**	***p*** < .001*
Smoking (in the past 30 days)	0.133 (0.017–1.012)	*p* = .051	0.781 (0.317–1.924)	*p* = .591
Alcohol drinking (in the past 30 days)	0.641 (0.366–1.123)	*p* = .120	1.459 (0.927–2.296)	*p* = .103

	FAS score^a^ (medium vs. low [reference group])		FAS score^a^ (high vs. low [reference group])	
Overall good health (self‐perceived)	1.081 (0.737–1.584)	*p* = .691	1.262 (0.340‐4.677)	*p* = .728
Poor hygiene	1.040 (0.716–1.511)	*p* = .838	1.477 (0.415–5.249)	*p* = .547
Insufficient intake of vegetables	0.884 (0.538–‐1.453)	*p* = .627	0.410 (0.119–1.410)	*p* = .157
Insufficient intake of fruits	0.901 (0.636–1.275)	*p* = .556	0.986 (0.309–3.144)	*p* = .981
Skipping breakfast	0.924 (0.644–1.325)	*p* = .666	0.784 (0.229–2.681)	*p* = .669
Physical inactivity	**0.612 (0.378–0.991)**	***p*** = .046*	1.598 (0.412–6.191)	*p* = .498
Spending >2 h on TV	1.162 (0.798–1.692)	*p* = .434	0.401 (0.124–1.292)	*p* = .126
Spending >2 h on games	1.124 (0.787–1.606)	*p* = .521	0.467 (0.136–1.598)	*p* = .225
Spending >2 h on social media	1.395 (0.977–1.992)	*p* = .067	0.872 (0.252–3.016)	*p* = .829
Smoking (in the past 30 days)	1.550 (0.577–4.166)	*p* = .385	5.119 (0.574–45.665)	*p* = .144
Alcohol drinking (in the past 30 days)	1.301 (0.782–2.164)	*p* = .310	1.820 (0.383–8.647)	*p* = .451

*Note*: Bold values are statistically significant *p* < 0.05.

Abbreviations: aOR, adjusted odds ratio; FAS, Family Affluence Scale.

^a^ aORs are adjusted for health literacy level, gender and FAS score.

*p < 0.05.

## 4 DISCUSSION

### 4.1 Summary of major findings

This study is a multifaceted study on the assessment of the health literacy level among Hong Kong adolescents and its association with various health behaviours. The major findings include: (1) overall, the majority of the adolescents in Hong Kong have limited health literacy, particularly in the domain of communication; (2) participation in the GoSmart Channel was significantly associated with improved adolescents' health literacy in almost all aspect; (3) desired level of health literacy was significantly associated with better perceived health and negatively associated with a range of unhealthy and risky behaviours including unhealthy dietary, poor hygiene, physical inactivity, smoking and alcohol consumption; and (4) the majority of adolescents relied on their parents for health information.

### 4.2 Source of health information

It was found that adolescents often rely on their relatives as a primary source of health information. Parents were identified to be the health behavioural models and the key role of adolescents' health decisions.^15^ They might encourage and discourage health behaviours through various means to prevent and reduce exposure to health risks.^16^, ^17^ However, the information may not always be reliable and accurate,^18^ and it heavily depends on the health literacy and lifestyle habits of the parents. Social media has become increasingly popular among adolescents as a source of health information; however, it also poses a risk of false information.^19^ Therefore, it is important to ensure reliability of the information source. For instance, the GoSmart Channel and websites of academic institutions provide credible and accurate information on health‐related topics. Promotion of these sources may enable access to trustworthy information among adolescents, allowing them to make informed decisions about their health.

### 4.3 Health literacy

Previous studies have evaluated health literacy among adolescents of different age cohorts in various countries: 22.7% of adolescent respondents aged 14–17 in Germany had a low level of health knowledge,^20^ while approximately 30%–40% of university students in Taiwan had limited health literacy.^7^ In this study, we found a surprisingly high proportion of adolescents in Hong Kong (74.4%) with limited health literacy, with the lowest level of knowledge in the communication subscale. It indicated that adolescents had inadequate or problematic ability to communicate with healthcare practitioners about their personal information, previous medications and questions they may have. The findings also demonstrated that participants faced challenges in sharing and obtaining health‐related information from their families and peers.

Similar to previous findings, our results identified higher SES as a possible factor associated with desired level of health literacy.^21^ However, most of these associations were insignificant due to the smaller sample size of participants with higher SES partly because of the travel restrictions during the coronavirus disease 2019 (COVID‐19) pandemic in this study.

### 4.4 Intervention

This study identified a significant association between the use of technology‐based intervention and the achievement of desired levels of health literacy among adolescents in almost every aspect. One of the explanations behind the success could be the accessibility of e‐learning, which allows learners to access relevant and up‐to‐date information from anywhere at any time, overcoming the physical obstacles, especially during the COVID‐19 pandemic.^22^, ^23^ Our findings are consistent with previous studies, which have shown that technology‐based health literacy interventions can significantly improve knowledge and awareness on various health issues.^24^, ^25^, ^26^ However, previous studies on interventions were not specifically focussed on adolescents, which makes our study particularly relevant. Nevertheless, follow‐up studies should be done to evaluate the sustainability and scalability of our intervention.

### 4.5 Association between health literacy and unhealthy behaviours

A large‐scale population‐based study in Taiwan has demonstrated a strong positive association between health literacy and obesity.^27^ Students with the highest health literacy were associated with a lower likelihood of being obese after controlling for sex and various factors.^27^ Our studies identified significant negative associations between higher health literacy and insufficient intake of fruits and vegetables. Furthermore, adolescents with higher health literacy were found significantly less likely to watch TV for more than 2 h and be physically inactive. As these factors were contributory to obesity (aOR_watch TV for more than 2 h_ = 1.55; 95% CI: 1.33–1.82, aOR_<5 fruits and vegetables_ = 1.35, 95% CI: 1.17–1.55; aOR_lack of physical exercise_ = 1.45, 95% CI: 1.15–2.12),^28^ our intervention may be effective in preventing obesity. Adolescents with desired health literacy were found marginally significantly less likely to engage in risky behaviours including alcohol drinking and smoking, which were consistent with prior studies.^15^, ^29^ A study in Denmark found that inadequate health literacy was associated with poor health status, inactivity and obesity, but to a lesser extent with smoking and alcohol consumption.^21^ A possible explanation was that poor health literacy was found associative with lower smoking risk knowledge and fewer negative smoking‐related attitudes.^30^ Likewise, adolescents with poor health literacy were more likely to be influenced by peers and relatives who drink.^31^

### 4.6 Limitations

The study has several limitations. First, the sample of participants may not be representative of the general population of adolescents in Hong Kong, which would limit the external validity of the study and may affect the generalizability of the results. Second, the use of self‐administered questionnaires, such as the HELMA questionnaire, may be subject to response bias or social desirability bias. Participants may provide answers that are socially acceptable rather than their true beliefs or behaviours, which may affect the validity of the results and may limit the accuracy of the data collected, especially as all data were based solely on adolescents' self‐reports. Cautions should be exercised when interpreting the results and drawing conclusions from the study. Lastly, it should be noted that due to the cross‐sectional nature of the study, the findings are considered hypothesis‐generating and no causal inference can be made. Further research should be done to establish a cause‐and‐effect relationship among the variables.

### 4.7 Conclusion

Overall, the low health literacy level found among Hong Kong adolescents suggests a pressing need for effective interventions to improve health literacy, particularly as strong links were found between high health literacy levels and healthier lifestyles. Meanwhile, our study provides valuable insights into the potential of technology‐based intervention to enhance health literacy levels among adolescents. It is also believed that the intervention would provide a credible source of health information for adolescents. Nevertheless, further research is needed to evaluate the sustainability and scalability of the intervention.

## CONFLICT OF INTEREST STATEMENT

The authors declare no conflict of interest.

## ETHICS STATEMENT

This study was approved by Survey and Behavioural Research Ethics (No. SBRE‐21‐0052), The Chinese University of Hong Kong, Hong Kong SAR. Our results will be disseminated through media outlets and presentations at scientific conferences and academic events.

## Supporting information

Supporting information.

Supporting information.

Supporting information.

## Data Availability

The data that supports the findings of this study are available from the corresponding author upon reasonable request.
