# Supplementary material for: Associations between GoSmart Channel, health literacy and health behaviours in adolescents: A population‐based study
Source: Health Expect. 2023 Oct 26;27(1):e13894. doi: 10.1111/hex.13894 (PMC10726208; doi:10.1111/hex.13894)
Supplement: Supplementary file 1 — Supporting information. [file HEX-27-e13894-s001.docx]

**Supplementary Tables Legends**

**Supplementary Table 1.** Source of health information

**Supplementary Table 2a.** Mean score of HELMA questionnaire

**Supplementary Table 2b.** Proportion of desired level of health literacy

**Supplementary Table 3.** Proportion of health behaviours by health literacy level

**Supplementary Table 1. Source of health information**

|  | n (%) |
| --- | --- |
| Relatives | 482 (63.7%) |
| School programme (Health-related topics) | 426 (56.2%) |
| TV programme or other videos (e.g.: youtube) | 417 (55.3%) |
| Social Media Platform (Facebook, Whatsapp, Instagram, etc.) | 368 (48.6%) |
| Teachers | 333 (44.0%) |
| Activities organized by schools | 327 (43.3%) |
| Medical professionals | 308 (40.6%) |
| Health-related app (e.g.: app for recording physical activity level) | 270 (35.7%) |
| Community Health Promotion activities, exhibitions or talks | 263 (34.9%) |
| Governmental website (CHP/ FHB) | 232 (30.6%) |
| Peers at school | 227 (29.9%) |
| Books (printed/ digital) | 221 (29.2%) |
| Websites of academic institution or organization | 204 (26.9%) |
| Newspaper or magazines (printed/ digital) | 200 (26.4%) |
| Email/ electronic newsletter | 177 (23.4%) |
| Personal website (sharing personal experience and perception) | 155 (20.6%) |
| Commercial website (introducing health product and service) | 142 (18.8%) |
| GoSmart Channel | 86 (11.4%) |

**Supplementary Table 2a – Mean score of HELMA questionnaire**

| Mean (sd) | Overall | GoSmart group | Control group | p |
| --- | --- | --- | --- | --- |
| Overall | 54.98 (17.39) | 61.29 (19.77) | 54.21 (16.77) | <0.001* |
| Access | 54.21 (22.26) | 63.55 (22.50) | 53.05 (21.91) | <0.001* |
| Reading | 52.66 (23.80) | 59.13 (25.24) | 51.87 (23.49) | 0.013* |
| Understanding | 60.68 (21.33) | 64.94 (22.35) | 60.26 (21.05) | 0.069 |
| Appraisal | 57.59 (21.65) | 65.53 (21.05) | 56.70 (21.45) | <0.001* |
| Use | 52.06 (22.64) | 61.41 (23.33) | 50.71 (22.26) | <0.001* |
| Communication | 51.82 (21.09) | 62.35 (22.48) | 50.40 (20.35) | <0.001* |
| Self-Efficacy | 51.85 (20.42) | 59.04 (22.52) | 50.89 (19.74) | <0.001* |
| Numeracy | 59.95 (33.98) | 53.33 (34.24) | 61.06 (34.02) | 0.078 |

**Supplementary Table 2b – Proportion of desired level of health literacy**

| % of desired level (n) | Overall | GoSmart group | Control group | p |
| --- | --- | --- | --- | --- |
| Overall | 25.6% (196) | 41.9% (36) | 23.6% (157) | <0.001* |
| Access | 28.6% (221) | 41.9% (36) | 27.2% (181) | 0.005* |
| Reading | 27.3% (210) | 36.0% (31) | 26.6% (176) | 0.065 |
| Understanding | 41.1% (312) | 46.5% (40) | 40.7% (266) | 0.301 |
| Appraisal | 33.7% (257) | 43.5% (37) | 32.8% (216) | 0.049* |
| Use | 25.8% (198) | 37.2% (32) | 24.3% (161) | 0.010* |
| Communication | 22.0% (166) | 38.8% (33) | 19.6% (129) | <0.001* |
| Self-Efficacy | 22.7% (175) | 35.3% (30) | 20.9% (139) | 0.003* |
| Numeracy | 54.2% (352) | 48.6% (34) | 55.3% (316) | 0.283 |

**Supplementary Table 3 – Proportion of health behaviours by health literacy level**

| % (n) | Desirable health literacy | Limited health literacy | p |
| --- | --- | --- | --- |
| Overall good health | 81.1% (159) | 67.6% (384) | <0.001* |
| Poor hygiene | 21.2% (40) | 35.5% (193) | <0.001* |
| Insufficient intake of vegetables | 77.5% (145) | 88.7% (495) | <0.001* |
| Insufficient intake of fruits | 43.1% (81) | 56.0% (309) | 0.002* |
| Skipping breakfast | 34.9% (65) | 45.6% (234) | 0.012* |
| Physical inactivity | 14.2% (27) | 22.2% (123) | 0.018* |
| Spending more than 2 hours on TV | 59.8% (113) | 68.6% (376) | 0.027* |
| Spending more than 2 hours on games | 43.3% (81) | 57.7% (315) | 0.001* |
| Spending more than 2 hours on social media | 36.4% (68) | 46.5% (256) | 0.016* |
| Smoking (past 30 days) | 0.5% (1) | 3.9% (21) | 0.021* |
| Alcohol consumption (past 30 days) | 9.1% (17) | 13.8% (75) | 0.092 |
