# Supplementary material for: Associations between GoSmart Channel, health literacy and health behaviours in adolescents: A population‐based study
Source: Health Expect. 2023 Oct 26;27(1):e13894. doi: 10.1111/hex.13894 (PMC10726208; doi:10.1111/hex.13894)
Supplement: Supplementary file 2 — Supporting information. [file HEX-27-e13894-s002.pdf]

Supplementary Material 1. Manual for scoring the HELMA

	**Number of items**	**Minimum possible raw score**	**Maximum possible raw score**
**Access**	**5 (item 5-9)**	**5**	**25**
**Reading**	**5 (item 10-14)**	**5**	**25**
**Understanding**	**10 (item 15-24)**	**10**	**50**
**Appraisal**	**5 (item 25-29)**	**5**	**25**
**Use**	**4 (item 30-33)**	**4**	**20**
**Communication**	**8 (item 34-41)**	**8**	**40**
**Self-efficacy**	**4 (item 1-4)**	**4**	**20**
**Numeracy**	**3 (item 42-44)**	**3**	**15**

To calculate each subscale or total score for the HELMA, first we added raw scores and linearly transferred it to a score from 0 to 100 using the following formula.

Score= $$\frac{Raw\ score - Minimum\ possible\ raw\ score}{Maximum\ possible\ raw\ score - Minimum\ possible\ raw\ score} * 100$$

We ranked the HELMA score to 4 categories: ‘inadequate’, ‘problematic’ (which together also define ‘limited’ health literacy), ‘sufficient and ‘excellent’ (which together also defined ‘desired’ health literacy):
Inadequate= 0–50
Problematic= 50.1–66
Sufficient= 66.1–84
Excellent=84.1-100
