# Supplementary material for: Associations between GoSmart Channel, health literacy and health behaviours in adolescents: A population‐based study
Source: Health Expect. 2023 Oct 26;27(1):e13894. doi: 10.1111/hex.13894 (PMC10726208; doi:10.1111/hex.13894)
Supplement: Supplementary file 3 — Supporting information. [file HEX-27-e13894-s003.pdf]

Supplementary Material 2. Questionnaire

GoSmart.Net 深化計劃

健康素養與生活習慣問卷調查（2021-22 年度）

本問卷的目的是評估青少年的健康素養水平，並了解可能影響青少年的健康行為和想法。你和所有參與調查的學生所提供的資料將有助健康教育的未來發展。所以，我們需要你的參與，讓我們獲取更準確的資料。

請不要在問卷填上姓名，學號只用來配對跟進的問卷。本調查所得的資料只用作統計用途，問卷亦只會由香港中文大學的研究人員翻閱和保密處理。那些有關個人和家庭背景的資料只用來反映參與這次調查的學生之整體背景，並不會用來辨認你的身份。調查結果亦不會影響你的校內表現或成績。**你只需依據實際行為作答。**如果你不願意回答某些問題，讓它留空便可。

請用黑色原子筆填寫，把所選答案相應的圓格填滿（○ → ●），並在方格內提供所需資料。除特別說明外，每題只可選一個答案。感謝你的參與！

香港中文大學醫學院
賽馬會公共衛生及基層醫療學院
健康教育及促進健康中心

- 以下問題關於你的基本資料：

1. 你目前就讀的班別：________________

2. 你的學號：________________

3. 你的出生日期：______________年______月_______日

4. 你的性別： ○ 男 ○ 女

5. **在過去 30 天內**，你覺得你的健康情況大概是：

○ 很好
○ 好
○ 普通
○ 差
○ 很差

6. 你的家庭有沒有擁有私家車或其他車輛?

- O 沒有
- O 有，一部
- O 有，兩部或以上

7. 你有沒有屬於自己的睡房？（不包括上下格床或與其他家庭成員同房）

O 沒有　　O 有

8. 在過去的 12 個月內，你有沒有與家人在放假時離開香港旅行？（不包括純粹回鄉探親、掃墓）

O 沒有　　O 兩次
O 一次　　O 三次或以上

9. 你家裏有多少部電腦？（包括家用桌上電腦、手提電腦及平板電腦，但不包括智能電話）

O 沒有　　O 兩部
O 一部　　O 三部或以上

▪ 以下問題關於你覺得自己在獲取和使用健康資訊方面的能力和才幹。請就以下每條關於你的能力或技能的描述，選出最貼切的選項，並回答所有題目。

描述	從不	很少	有時	通常	總是
10. 我盡可能嘗試獲取多些有關健康的資訊。I try to get more information about health as much as possible	O	O	O	O	O
11. 我能夠找到我需要的健康資訊。I am able to find health information that I need	O	O	O	O	O
12. 當生病或面對健康問題時，我能獲取我所需的必要資訊。When ill or facing health problems, I can get the necessary information I need	O	O	O	O	O
13. 我能夠向他人詢問我需要的健康資訊。I am able to ask others about health information that I need	O	O	O	O	O
14. 我能夠接觸到關於切合我年齡層的健康飲食資訊。I am able to access information about the healthy diet that is appropriate for my age group	O	O	O	O	O
15. 我能夠接觸到關於切合我年齡層的體能活動資訊。I am able to access information about the physical activity appropriate for my age group	O	O	O	O	O
16. 我能夠接觸到關於我頭髮和皮膚所需的適當護理，且切合我年紀的相關資訊。I am able to access information about the proper care required for my skin and hair that is appropriate for my age group	O	O	O	O	O

描述	從不	很少	有時	通常	總是
17. 我能夠接觸到關於切合我這年齡層的心理健康資訊。I am able to access information about mental health appropriate for my age group	O	O	O	O	O
18. 我能夠從互聯網找到關於健康資訊的有用資源。I am able to find useful resources about health Information on the Internet	O	O	O	O	O
19. 我能讀懂有關處方藥物的小冊子。I can read brochures on prescribed medicine	O	O	O	O	O
20. 我能輕鬆地讀懂有關營養議題的教育小冊子。I can easily read educational brochures about nutritional issues	O	O	O	O	O
21. 我能輕鬆地讀懂有關疾病預防（例如貧血．骨質疏鬆症、呼吸道感染等）的小冊子/實況報道。I can easily read brochures/fact sheets about disease prevention (e.g. anaemia, osteoporosis, respiratory infections, etc.)	O	O	O	O	O
22. 我能輕鬆地讀懂雜誌和報章上的健康資訊素材。I can easily read health information materials in magazines and newspapers	O	O	O	O	O
23. 我能輕鬆地讀懂在互聯網（例如網站）上的健康資訊素材。I can easily read health information materials on the Internet (e.g. websites)	O	O	O	O	O
24. 對於醫院和醫務中心所用的標誌，我都能容易地理解其含義。I can easily understand the meaning of the signs used in hospitals and medical centres	O	O	O	O	O
25. 對於所聽到關於健康的事，我大多數都能理解。I can understand most things I hear about health	O	O	O	O	O
26. 對於所找到的健康資訊，我都能容易地理解其內容。I can easily understand the content of health information that I find	O	O	O	O	O
27. 對於我的醫生給予的指示和建議（例如處方），我都能容易地理解。I can easily understand my doctor's instructions and recommendations (e.g. prescriptions)	O	O	O	O	O
28. 對於有關藥物的資訊－用法、副作用和警告，我都能容易地理解。I can easily understand information about medications – usage, side effects and warnings	O	O	O	O	O
29. 對於食品包裝上的營養資料，我都能容易地理解。I can easily understand the nutrition facts on food packages	O	O	O	O	O
30. 對於媒體（如電台廣播、電視、互聯網等）上關於青少年適當營養的資訊和建議，我都能理解。I can understand the information and recommendations about proper nutrition for adolescents in the media (e.g. radio, TV, internet, etc.)	O	O	O	O	O
31. 對於媒體（如電台廣播、電視、互聯網等）提供有關煙草、藥物濫用和風險行為的資訊和警告，我都能理解。I can understand the information and warnings provided by the media (e.g. radio, TV, internet, etc.) about tobacco, drug abuse and risky behaviours	O	O	O	O	O

描述	從不	很少	有時	通常	總是
32. 對於媒體上有關健康與病患的資訊和建議，我都能理解。I can understand the information and recommendations about health and illness in the media	O	O	O	O	O
33. 對於有關預防意外與受傷的建議，我都能理解。I can understand the recommendations on prevention of accidents and injuries	O	O	O	O	O
34. 當面對新的健康資訊時，我能夠判斷其準確性。When faced with new health information, I can judge its accuracy	O	O	O	O	O
35. 對於從不同來源獲取的數據，我會加以比較。I would compare the data obtained from various sources	O	O	O	O	O
36. 當要處理有矛盾的健康資訊時，我都能辨別出正確的資訊。When dealing with conflicting information about health issues, I can recognize the correct information	O	O	O	O	O
37. 我有能力判斷哪些資源是我可以信任的。I have the ability to judge which resources I can trust	O	O	O	O	O
38. 當要處理有關營養的資訊時，我都能選擇正確的資訊。When dealing with nutritional information I can choose the right information	O	O	O	O	O
39. 當買東西時，我會根據包裝上所寫的營養資訊（例如能量、糖、蛋白質的分量等）來選購食物。When shopping, I choose food based on its nutrition facts (e.g. amount of energy, sugar, protein, etc.) written on the packaging	O	O	O	O	O
40. 我盡量選擇沒有防腐劑的食物。I try to choose foods without preservatives	O	O	O	O	O
41. 我嘗試將所學到關於健康的知識應用到日常生活中。I try to apply what I have learned about health issues in my everyday life	O	O	O	O	O
42. 我盡量保持體重處於平衡狀態。I try to keep my body weight in balance	O	O	O	O	O
43. 我能與醫護人員討論我對健康問題的擔憂。I can discuss my concerns relating to health issues with health providers	O	O	O	O	O
44. 在看醫生（或其他醫護人員）的時候，我能夠給予他（或她）全部必要關於我的個人資料。When visiting a doctor or health provider I am able to give him/her all of my necessary personal information	O	O	O	O	O
45. 在看醫生（或其他醫護人員）的時候，我能夠告訴他（或她）我先前用過的藥物名稱。When visiting a doctor or health provider I am able to tell him/her the name of the medications that I have previously used	O	O	O	O	O
46. 在看醫生（或其他醫護人員）的時候，我能夠詢問我有的一切問題。When visiting a doctor or health provider I am able to ask all the questions I have	O	O	O	O	O

描述	從不	很少	有時	通常	總是
47. 我能與他人（例如家人、朋友等）分享我所收集的健康資訊。I can share the health information that I gather with others (e.g. family, friends, etc.)	○	○	○	○	○
48. 若我對健康有任何疑問，我能夠從他人獲得資訊和建議。If I have any questions about health issues I am able to get information and advice from others	○	○	○	○	○
49. 在看醫生（或醫護人員）的時候，我能夠根據自己的研究來提出問題。When visiting a doctor or health provide I am able to ask questions based on my research	○	○	○	○	○
50. 我會與朋友談到有關避免風險行為（例如吸煙、水煙、毒品等）。I talk to my friends about avoiding risky behaviour (e.g. smoking, hookah, drugs, etc.)	○	○	○	○	○

51. 在牛奶容器的背面有這些資料。假設某人一天喝了三杯牛奶，他（或她）共攝取了多少碳水化合物呢？

答案：

營養資料 Nutrition Facts
食用份量 Serving size：1 杯 cup（240 毫升 cc）
此容器共有 Servings per container：4 份
每份含有 Amount per serving：
能量 Energy：140 千卡 Kcal
總脂肪 Total Fat：7 克 g
膽固醇 Cholesterol: 30 毫克 mg
碳水化合物 Carbohydrates：11 克 g
糖 Sugar: 0 克 g
蛋白質 Protein：8 克 g
鈉 Sodium：160 毫克 mg

52. 試計算一位身高 160 厘米，體重 70 公斤人士的體質指數（BMI）？

答案：

（準確至小數後一個位）

$$\text{體質指數} = \frac{\text{體重(公斤)}}{\text{身高(米)}^2}$$

$$\text{BMI} = \frac{weight(kg)}{height(m)^2}$$

53. 承上題，這位人士的肥胖狀況為何（根據右表的資訊）？

a. 過輕
b. 體重正常
c. 超重
d. 肥胖

答案：

（填上代表的字母）

	體質指數 BMI
過輕 Underweight	<18.5
體重正常 Normal weight	18.5-24.9
超重 Overweight	25-29.9
肥胖 Obese	≥30

Source: Ghanbari, S., Ramezankhani, A., Montazeri, A., & Mehrabi, Y. (2016). Health literacy measure for adolescents (HELMA): Development and psychometric properties. *PloS one, 11*(2), e0149202. doi: 10.1371/journal.pone.0149202

▪ 以下問題關於你覺得自己在獲取和使用健康資訊方面的能力和才幹。請就以下每條關於你的能力或技能的描述，選出最貼切的選項，並回答所有題目。

**你**有多麼容易或困難去......<br>How easy or difficult is it **for you** to…	非常困難	困難	容易	非常容易
54 . . . 找出如何令你在感冒時能快些痊癒？find out how to recover quickly when you have a cold?	O	O	O	O
55 . . . 找出可以做什麼來令你不會太胖或太瘦？find out what you can do so that you don't get too fat or too thin?	O	O	O	O
56 . . . 找出怎樣令你最有效地放鬆自己？find out how you can best relax?	O	O	O	O
57 . . . 找出哪種食物對你有益？find out which food is healthy for you?	O	O	O	O
58 . . . . 明白當你患病時該在何時服藥和怎樣服藥？understand when and how you should take your medicine when you are ill?	O	O	O	O
59 . . . . 明白醫生對你說的話？understand what your doctor says to you?	O	O	O	O
60 . . . 明白為甚麼即使你沒有生病，有時也有需要看醫生？understand why you sometimes need to see the doctor even though you are not ill?	O	O	O	O
61 . . . 明白為甚麼需要接種疫苗？understand why you need vaccinations?	O	O	O	O
62 . . . 明白父母對你說關於你健康的事？understand what your parents tell you about your health?	O	O	O	O
63. ……明白為甚麼你有時候需要放鬆一下？understand why you need to relax sometimes?	O	O	O	O
64. ……判斷有甚麼是對你保持健康很有幫忙，又有甚麼是沒有多大幫助的？judge what helps a lot for you to stay healthy and what does not help much?	O	O	O	O
65. ……（在患病時）照父母告訴你的去做，以致你可以康復過來？do what your parents tell you to do so that you can get well again?	O	O	O	O
66. ……（在患病時）遵照所吩咐的方法去服藥？take your medicine in the way you're told to?	O	O	O	O
67. ……遵守你從課堂所學到的道路安全知識？stick to what you have learned in road safety lessons?	O	O	O	O
68. ……吃得健康？have a healthy diet?	O	O	O	O

▪ 第 69 至 71 題，請按以下句子中，英文字母的位置挑選合適詞語，使句子的意思變得完整：

「要預防傳染病，我們需要維持良好的__A__習慣，還可以通過__B__疫苗來提升個人__C__。」

A)	B)	C)
○ 整理	○ 抗生素	○ 忍耐力
○ 衛生	○ 細菌	○ 免疫力
○ 閱讀	○ 消毒	○ 吸收力
○ 守時	○ 傳播	○ 消化力
○ 早餐	○ 接種	○ 感染力

「我們需要從不同食物攝取各種__D__。綠葉蔬菜含有豐富的__E__，有助維持健康。肉類則含有豐富的__F__，能滿足兒童的生長需要。」

D)	E)	F)
○ 鹽分	○ 添加劑	○ 防腐劑
○ 營養素	○ 能量	○ 糖分
○ 水分	○ 澱粉	○ 水分
○ 能量	○ 脂肪	○ 蛋白質
○ 碳水化合物	○ 維生素	○ 澱粉

「要保持理想的體重，我們需要維持均衡__G__，並要多做__H__，避免過胖或__I__。」

G)	H)	I)
○ 姿勢	○ 善事	○ 過瘦
○ 飲食	○ 家務	○ 過勞
○ 睡眠	○ 家課	○ 過界
○ 收入	○ 靜態活動	○ 過大
○ 工作	○ 運動	○ 過小

▪ 以下問題關於你對一些描述的看法。

72. 「我的父母確保我吃得健康」，你同意這描述嗎？

☐	☐	☐	☐
完全不同意	不同意	同意	完全同意

73.「父母認為我定期做運動是很重要的」，你同意這描述嗎？

☐	☐	☐	☐
完全不同意	不同意	同意	完全同意

74.「對於大多數問題，我都能找到解決方案」，這描述真確嗎？

☐	☐	☐	☐
根本不是真的	不是真的	相當真確	完全正確

75.「我喜歡學習有關健康的新知識」，這描述真確嗎？

根本不是真的	相當不真確	相當真確	完全正確

- 以下問題關於你獲取健康知識的途徑：

76. **在過去 12 個月**，你有沒有從以下途徑獲得**健康**方面的新知識？（每項途徑選一項）

	有	沒有
● 醫護人員（醫生、護士、治療師、註冊中醫師）	○	○
● 親友（包括父母、兄弟姊妹、朋友、鄰居）	○	○
● 校內師長（包括教師、教練、校護等）	○	○
● 校內朋輩（包括學生健康大使、健康軍團等）	○	○
● 學校課程（與健康相關的課題）	○	○
● 學校舉辦的健康活動	○	○
● 社區健康推廣活動、展覽、講座	○	○
● GoSmart Channel	○	○
● 電視節目和其他網上視訊（如 YouTube 影片或視頻）	○	○
● 社交應用程式（如 Facebook、WhatsApp、Instagram 等）	○	○
● 與健康相關的電子應用程式（如記錄運動量的程式）	○	○
● 書籍（電子或實體版）	○	○
● 報章、雜誌（電子或實體版）	○	○
● 電子郵件、電子通訊刊物	○	○
● 政府衞生部門網頁（如衞生防護中心、食環署網頁）	○	○
● 學術機構及組織的網頁	○	○
● 商務網頁（如介紹健康產品和服務的網頁）	○	○
● 個人網頁（如分享個人經驗和見解的網誌）	○	○
● 其他途徑（請說明）：		

- 以下問題關於你的衛生習慣：

77. 你有每天在以下時候**潔手**的習慣嗎？用水洗手或以酒精搓手液消毒雙手也包括在內。（可選多項）

- ○ 沒有固定的潔手習慣（**跳至第 58 題**）
- ○ 每次小便後
- ○ 每次大便後
- ○ 剛回家後
- ○ 每次看見手上有明顯污垢時
- ○ 每次食飯前
- ○ 每次擦眼睛或挖鼻孔之前
- ○ 每次擦眼睛或挖鼻孔之後
- ○ 其他時候：

78. 你怎樣形容你的**洗手**習慣？（選最貼切的一項）

- ○ **每次**都認真地用梘液搓手的每個部位，包括手背、指隙和手腕
- ○ **有時**會認真地用梘液搓手的每個部位，但並非每次都那麼認真
- ○ **有時**會草率地洗手，只用梘液搓手掌和手指
- ○ **很多時**草率地洗手，也不一定使用梘液
- ○ **沒有**固定的洗手習慣

79. 你有每天在以下時候**刷牙**的習慣嗎？（可選多項）

- ○ 沒有固定刷牙習慣（**跳至第 60 題**）
- ○ 每天起床後
- ○ 每天睡覺前
- ○ 每天早餐後
- ○ 每天午餐後
- ○ 每天晚餐後

80. 你怎樣形容你的**刷牙**習慣？（選最貼切的一項）

- ○ **每次**都認真地刷每顆牙的各個部位，包括外側面、內側面和咀嚼面
- ○ **有時**會認真地刷每顆牙的每個部位，不一定每次都刷到牙齒的內側面和口腔最入面的大牙
- ○ **有時**會草率地刷，可能很快便刷完
- ○ **時**草率地刷，通常很快便刷完
- ○ **沒有**固定的刷牙習慣

81. **在過去 7 天內**，你曾多少次用牙線清潔牙縫？

- ○ 0 次
- ○ 1 至 3 次
- ○ 4 至 6 次
- ○ 每天 1 次
- ○ 每天多於 1 次
- ○ 不知道牙線是甚麼

- 以下問題關於你的飲食習慣：

82. **在過去 7 天內**，你曾進食以下食物多少次？

食物（每次分量）	沒有	7 天內 1 至 3 次	7 天內 4 至 6 次	每天 1 次或以上
● 脆口零食，如薯片、蝦條等（每小包約 35 克）	○	○	○	○
● 朱古力或糖果（3 至 5 顆）	○	○	○	○
● 甜品、雪糕、蛋糕或西餅（每件或每杯約 150 克）	○	○	○	○
● 汽水（每罐約 330 毫升）	○	○	○	○
● 含糖飲品，如紙包果汁、檸檬茶等（每包或每杯約 250 毫升）	○	○	○	○
● 油炸食物，如炸薯條、炸雞等	○	○	○	○
● 加工或醃製肉類，如香腸、火腿、叉燒、臘腸等	○	○	○	○

83. **在過去 7 天內**，你平均每天進食多少瓜菜？

- ○ 完全沒有進食瓜菜
- ○ 偶爾進食一兩條瓜菜
- ○ 每天進食少於半碗瓜菜
- ○ 每天進食半碗瓜菜
- ○ 每天進食 1 平碗至不足 1.5 碗瓜菜
- ○ 每天進食 1.5 碗或更多的瓜菜
- ○ 不肯定

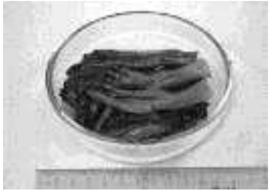

*中型碗參考

84. **在過去 7 天內**，你平均每天進食多少水果？每份水果大約相等於 1 個中型水果（如奇異果）、半個大型水果（如火龍果）或半碗水果（如葡萄）的分量。

- ○ 完全沒有進食水果
- ○ 偶爾進食一兩次
- ○ 每天半份水果
- ○ 每天 1 份水果
- ○ 每天 2 份水果或以上
- ○ 不肯定

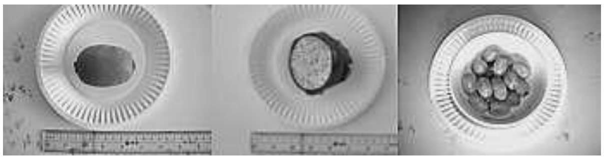

每份水果參考

85. **在過去 7 天內**，你在多少天有吃早餐？

- ○ 7 天都有吃早餐
- ○ 5 至 6 天
- ○ 3 至 4 天
- ○ 1 至 2 天
- ○ 0 天
- ○ 不肯定

▪ 以下問題關於你的體能活動。

86. **在過去 7 天內**，你有多少天曾進行累積至少 60 分鐘中等至劇烈強度的**體能活動**？

「中等強度」是指在活動時呼吸加快但仍能如常說話，如進行踏單車、急步行、乒乓球、 公園玩耍等，而「劇烈強度」是指在活動時呼吸急促得不能如常說話，如進行踢足球、跳繩、跑步、籃球等。舉例說，小明在體育課時打乒乓球 35 分鐘，放學後又打籃球 30 分鐘，全日合共 65 分鐘，可視為有一天能累積至少 60 分鐘中等至劇烈程度的**體能活動**。

- O 0 天
- O 1 天
- O 2 天
- O 3 天
- O 4 天
- O 5 天
- O 6 天
- O 每天

87. **在過去 7 天內**，你曾進行哪種**體能活動**？（可選多項）

- O 我在過去 7 天內沒有進行任何體能活動**（跳至第 68 題）**
- O 跑步、慢跑
- O 集體遊戲（如數字球、閃避球等）
- O 游泳、水上活動
- O 球類活動（如籃球、足球、排球等）
- O 跳繩
- O 體操、跳舞
- O 武術（如中國功夫、跆拳道、劍擊等）
- O 騎單車
- O 遠足、行山
- O 器械健身（包括健身單車、划艇機等）
- O 伸展和鍛煉（包括瑜伽、仰臥起坐等）
- O 溜冰或滾軸溜冰
- O 電子體感遊戲
- O 在遊樂場玩耍
- O 其他：

▪ 以下問題關於你的靜態活動習慣：

88. 一般來說，你每天累積多少時間看電視、網上電視節目、YouTube 影片呢？

a. 平常上課的日子（只選一項）：

- O 我**不會**在平常上課的日子看視訊
- O 每天少於 1 小時
- O 每天 1 小時至不足 2 小時
- O 每天 2 小時至不足 3 小時
- O 每天 3 小時至不足 4 小時
- O 每天 4 小時至不足 5 小時
- O 每天 5 小時至不足 6 小時
- O 每天 6 小時至不足 7 小時
- O 每天 7 小時至不足 8 小時
- O 每天 8 小時或以上

b. 假日（只選一項）：

- ○ 我**不會**在**假日**看視訊
- ○ 每天少於 1 小時
- ○ 每天 1 小時至不足 2 小時
- ○ 每天 2 小時至不足 3 小時
- ○ 每天 3 小時至不足 4 小時
- ○ 每天 4 小時至不足 5 小時
- ○ 每天 5 小時至不足 6 小時
- ○ 每天 6 小時至不足 7 小時
- ○ 每天 7 小時至不足 8 小時
- ○ 每天 8 小時或以上

89. 一般來說，你每天累積多少時間玩電子或電腦遊戲？（媒體可包括遊戲機、智能電 話、平板電腦及桌上電腦等）

a. 平常上課的日子（選以下其中一項）：

- ○ 我**不會**在平常上課的日子打機及只會因做功課而使用電腦
- ○ 每天少於 1 小時
- ○ 每天 1 小時至不足 2 小時
- ○ 每天 2 小時至不足 3 小時
- ○ 每天 3 小時至不足 4 小時
- ○ 每天 4 小時至不足 5 小時
- ○ 每天 5 小時至不足 6 小時
- ○ 每天 6 小時至不足 7 小時
- ○ 每天 7 小時至不足 8 小時
- ○ 每天 8 小時或以上

b. 假日（選以下其中一項）：

- ○ 我**不會**在**假日**打機及只會因學業而使用電腦
- ○ 每天少於 1 小時
- ○ 每天 1 小時至不足 2 小時
- ○ 每天 2 小時至不足 3 小時
- ○ 每天 3 小時至不足 4 小時
- ○ 每天 4 小時至不足 5 小時
- ○ 每天 5 小時至不足 6 小時
- ○ 每天 6 小時至不足 7 小時
- ○ 每天 7 小時至不足 8 小時
- ○ 每天 8 小時或以上

90. 一般來說，你每天累積多少時間使用社交應用程式或瀏覽社交網站？（如 Facebook、Instagram、WhatsApp、微信、微博或網誌）

a. 平常上課的日子（選以下其中一項）：

- ○ 我**不會**在平常上課的日子使用社交應用程式或瀏覽社交網站
- ○ 每天少於 1 小時
- ○ 每天 1 小時至不足 2 小時
- ○ 每天 2 小時至不足 3 小時
- ○ 每天 3 小時至不足 4 小時
- ○ 每天 4 小時至不足 5 小時
- ○ 每天 5 小時至不足 6 小時
- ○ 每天 6 小時至不足 7 小時
- ○ 每天 7 小時至不足 8 小時
- ○ 每天 8 小時或以上

b. **假日（選以下其中一項）**：

- ○ 我**不會**在**假日**使用社交應用程式或瀏覽社交網站
- ○ 每天少於 1 小時
- ○ 每天 1 小時至不足 2 小時
- ○ 每天 2 小時至不足 3 小時
- ○ 每天 3 小時至不足 4 小時
- ○ 每天 4 小時至不足 5 小時
- ○ 每天 5 小時至不足 6 小時
- ○ 每天 6 小時至不足 7 小時
- ○ 每天 7 小時至不足 8 小時
- ○ 每天 8 小時或以上

91. 你認為上述活動（看電視和網上視訊、玩遊戲機、使用社交應用程式或瀏覽社交網站） 對你現時日常生活及健康造成甚麼影響呢？ （可選多項）

- ○ 沒有影響**（跳至第 72 題）**
- ○ 眼睛疲倦或不適
- ○ 手部不適
- ○ 腰背不適
- ○ 肩頸不適
- ○ 與家人爭吵
- ○ 睡眠不足導致身體疲倦
- ○ 令情緒出現起伏
- ○ 影響學習
- ○ 集中力下降
- ○ 近視加深
- ○ 其他影響：

▪ 以下問題關於你過去一個月的平常睡眠習慣。你的回答應該能最準確反映過去一個月內大多數白天和夜間的情況。

92. **在過去一個月內**，你晚上通常甚麼時候上床睡覺？

- ○ 晚上 9:00
- ○ 晚上 9:30
- ○ 晚上 10:00
- ○ 晚上 10:30
- ○ 晚上 11:00
- ○ 晚上 11:30
- ○ 凌晨 12:00
- ○ 凌晨 12:30
- ○ 凌晨 1:00
- ○ 凌晨 1:30
- ○ 其他時間：

93. **在過去一個月內**，你通常在早上甚麼時候起床？

- ○ 早上 5:00
- ○ 早上 5:30
- ○ 早上 6:00
- ○ 早上 6:30
- ○ 早上 7:00
- ○ 早上 7:30
- ○ 早上 8:00
- ○ 早上 8:30
- ○ 其他時間：

▪ 以下問題關於你對自己體重的看法。

94. 你怎樣形容自己現時的體重？

- ○ 非常過輕
- ○ 少少過輕
- ○ 正常
- ○ 少少過重
- ○ 非常過重

95. 對於你的體重，你正嘗試：

- ○ 我**不會**為我的體重**做任何事情**
- ○ **減重**
- ○ **保持**現時的體重
- ○ **增加**體重

▪ 以下問題關於吸煙、飲酒和濫藥情況。你所填的答案不會被研究人員以外的人查閱。

96. 你曾試過吸煙嗎？（只吸一兩口也計算在內）

- ○ 從未試過
- ○ 曾經試過

97. 在過去 30 天內，你有多少天曾吸煙？

- ○ 沒有吸煙
- ○ 1 至 2 天
- ○ 3 至 5 天
- ○ 6 至 9 天
- ○ 10 至 19 天
- ○ 20 至 29 天
- ○ 每天

98. 你曾試過飲酒嗎？（只試過飲一兩口，或飲用含酒精的汽水<u>不計算</u>在內）

- ○ 從未試過
- ○ 曾經試過

99. 在過去 30 天內，你有多少天曾喝至少一杯酒？（如啤酒、紅酒、白酒）？

- ○ 沒有喝酒
- ○ 1 至 2 天
- ○ 3 至 5 天
- ○ 6 至 9 天
- ○ 10 至 19 天
- ○ 20 至 29 天
- ○ 每天

100. 你曾試過濫用藥物嗎？（如氯胺酮、搖頭丸、有機溶劑、海洛英等，但<u>不包括</u>醫生處 方給你的藥物）

- ○ 從未試過
- ○ 曾經試過

~問卷完~
